# Supplementary figures and images for: Microbiota composition of Culex perexiguus mosquitoes during the West Nile virus outbreak in southern Spain
Source: PLoS One. 2024 Nov 18;19(11):e0314001. doi: 10.1371/journal.pone.0314001 (PMC11573153; doi:10.1371/journal.pone.0314001)

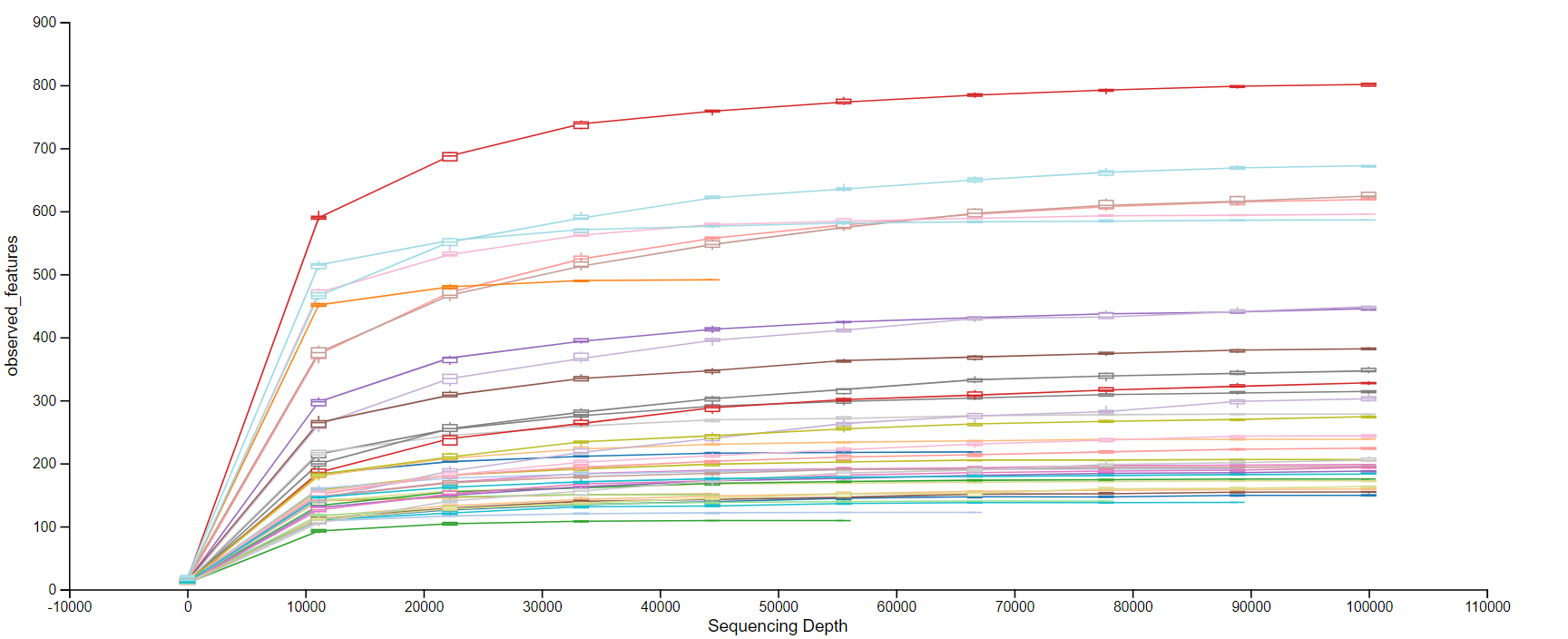

Supplement: S1 Fig — Rarefaction of the samples consists in randomly keep a specific number of sequencing reads from the sample, removing the rest of the reads. The rarefaction curve represents the number of ASVs present in each sample (y-axis) when rarified to different number of reads (x-axis). If a sample plateau, it indicates that its sequencing depth was sufficient to represent the bacterial diversity in that sample. (PNG) [file pone.0314001.s001.png]

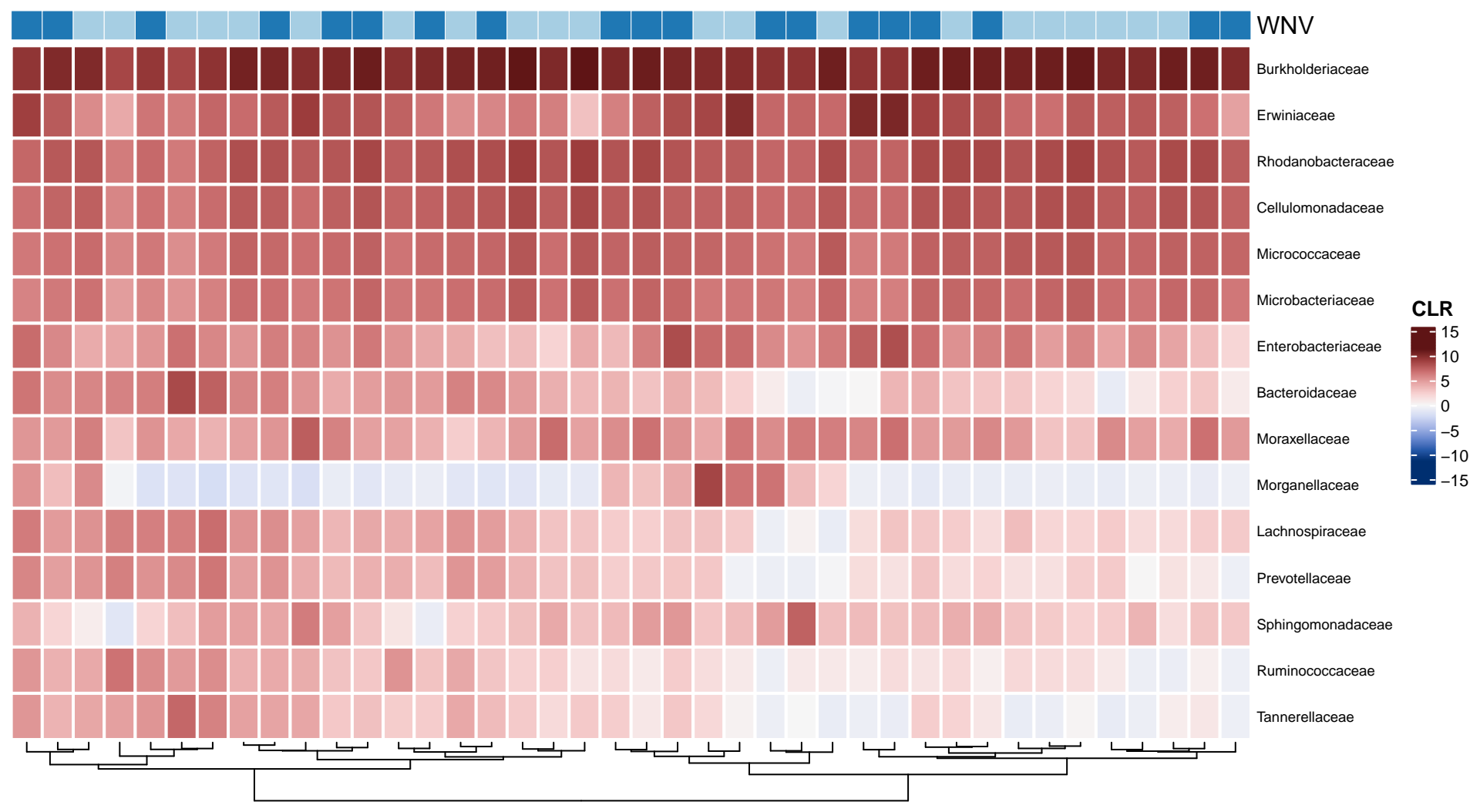

Supplement: S2 Fig — The figure shows the 15 most abundant families. Higher CLR values are colored red and correspond to higher relative abundances, while lower CLR values are colored blue and correspond to lower relative abundances. In the legend above the graph blue corresponds to WNV-positive samples and light blue to WNV-negative samples. The tree below the graph shows the samples grouped according to the similarity of microbiota composition based on Euclidean distances. (PDF) [file pone.0314001.s002.pdf]

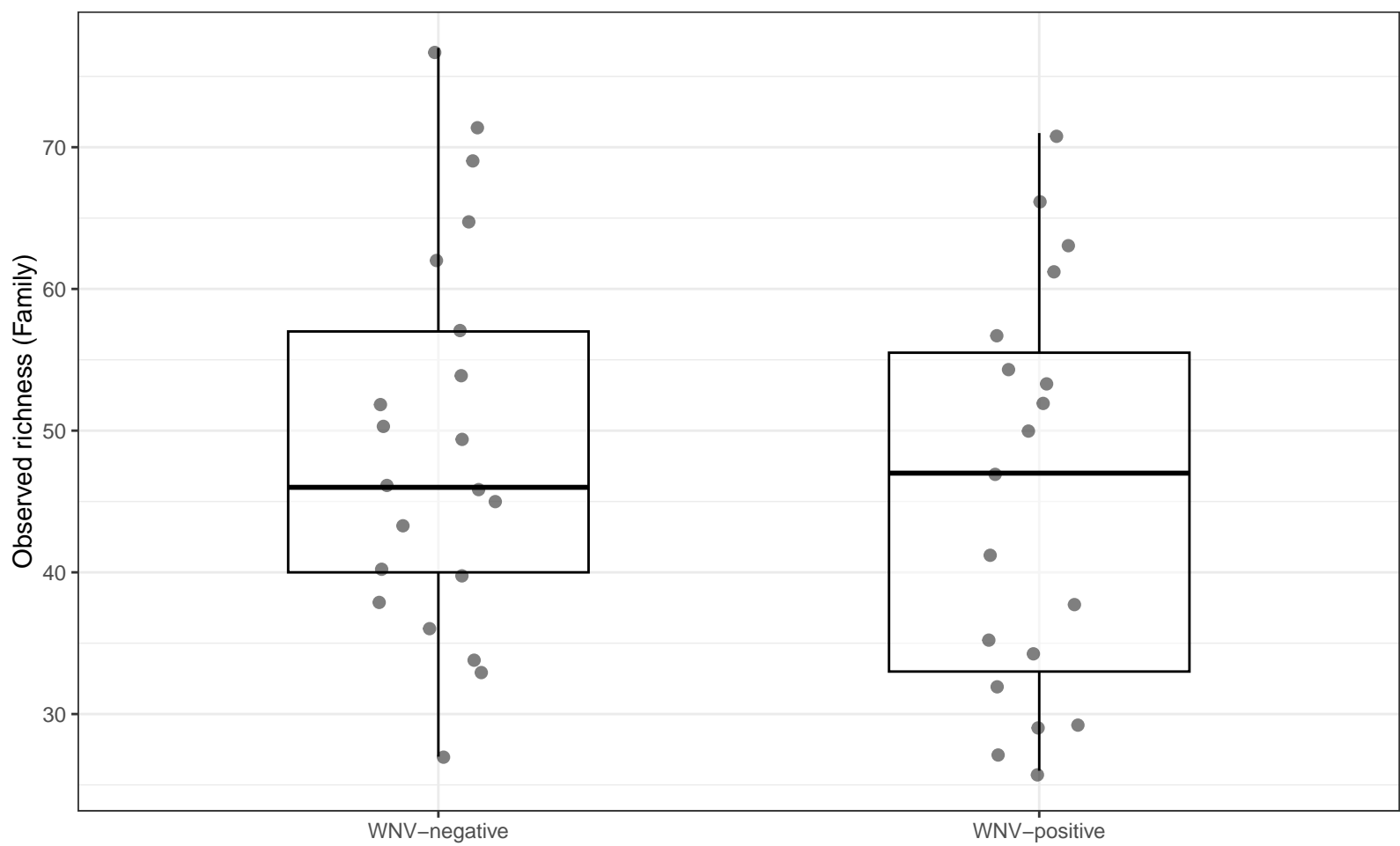

Supplement: S3 Fig — The vertical lines go from the lower and upper quartiles to the minimum or maximum, respectively, and the horizontal line represents the median. (PDF) [file pone.0314001.s003.pdf]

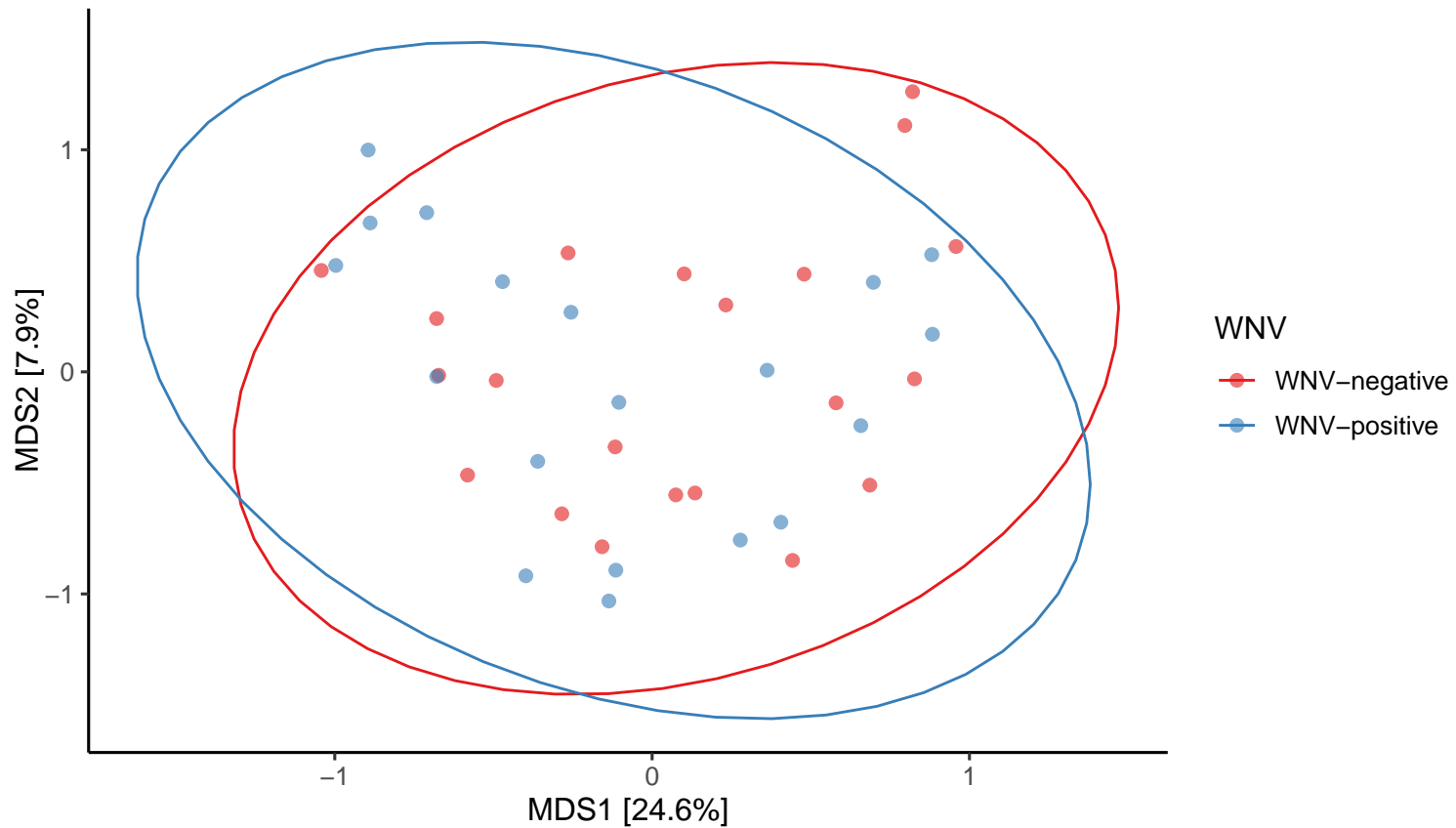

Supplement: S4 Fig — The percentage of variation explained by each component (axis) is shown in square brackets. (PDF) [file pone.0314001.s004.pdf]
