# Supplementary material for: Microbiota composition of Culex perexiguus mosquitoes during the West Nile virus outbreak in southern Spain
Source: PLoS One. 2024 Nov 18;19(11):e0314001. doi: 10.1371/journal.pone.0314001 (PMC11573153; doi:10.1371/journal.pone.0314001)
Supplement: S1 File — (DOCX) [file pone.0314001.s005.docx]

#!/bin/bash

# Activate conda environment with qimme2

source /path/anaconda3/bin/activate

conda activate qiime2-2023.5

#Import data

qiime tools import --type 'SampleData[SequencesWithQuality]' --input-path se-manifest.txt --output-path 01_demux/Single-end-sequences-demux.qza --input-format SingleEndFastqManifestPhred33

## se-manifest.txt is a .txt file with the name of each sample, its complete path and its sequence direction.

# Visualize data

qiime demux summarize --i-data 01_demux/Single-end-sequences-demux.qza --o-visualization 01_demux/Single-end-sequences-demux.qzv

# Denoising (DADA2)

qiime dada2 denoise-pyro --i-demultiplexed-seqs 01_demux/Single-end-sequences-demux.qza --p-trim-left 15 --p-trunc-len 0 --output-dir 02_dada/mosquitos_WNV_dada2 --p-n-threads 0 --verbose

## This command generates three outputs to be visualized

# 1. Representative sequences: Identified ASVs

qiime feature-table tabulate-seqs --i-data 02_dada/mosquitos_WNV_dada2/representative_sequences.qza --o-visualization 02_dada/mosquitos_WNV_dada2/representative_sequences.qzv

# 2. Frequency table

qiime feature-table summarize --i-table 02_dada/mosquitos_WNV_dada2/table.qza --output-dir 02_dada/mosquitos_WNV_dada2/prueba --o-visualization 02_dada/mosquitos_WNV_dada2/table.qzv

# 3. denoising stats

qiimef tools export --input-path 02_dada/mosquitos_WNV_dada2/denoising_stats.qza --output-path 02_dada/mosquitos_WNV_dada2/denoising_stats.qzv

## Taxonomy identification

qiime feature-classifier classify-consensus-vsearch --i-query 02_dada/mosquitos_WNV_dada2/representative_sequences.qza --i-reference-reads database/silva/silva-138-99-seqs.qza --i-reference-taxonomy database/silva/silva-138-99-tax.qza --p-perc-identity 0.99 --o-classification table_casif_99_silva_vsearch.qza --output-dir 03_clasif_silva_99 --verbose

# database files are downloaded from SILVA webpage

## Transform the qiime2 artifact to BIOM format and generate taxonomy file

cd 03_clasif_silva_99

qiime tools export --input-path table_casif_99_silva_vsearch.qza --output-path ./

sed -e 's/Feature /#OTU /' -e 's/Taxon/taxonomy/' -e 's/Consensus/confidence/' taxonomy.tsv > taxonomy_bacteria.tsv

qiime tools export --input-path ../02_dada/mosquitos_WNV_dada2/table.qza --output-path ../Table_ASVs/

cd ../Table_ASVs/

biom add-metadata -i feature-table.biom -o asv-table_with_tax.biom --observation-metadata-fp ../03_clasif_silva_99/taxonomy_bacteria.tsv --sc-separated taxonomy

biom convert -i asv-table_with_tax.biom -o asv-table_with_tax.tsv --to-tsv --header-key taxonomy --table-type "OTU table"

# Filtering

## Remove singletons

mkdir 01_No_singletons

qiime feature-table filter-features --i-table ../02_dada/mosquitos_WNV_dada2/table.qza --p-min-frequency 2 --o-filtered-table 01_No_singletons/asv.table_nosingletons.qza

qiime tools export --input-path 01_No_singletons/asv.table_nosingletons.qza --output-path 01_No_singletons/ #this command generates feature-table.biom

biom convert -i 01_No_singletons/feature-table.biom -o 01_No_singletons/asv.table_nosingletons.txt --to-tsv --header-key taxonomy --table-type "OTU table"

biom summarize-table -i 01_No_singletons/feature-table.biom -o 01_No_singletons/asv-table_nosingletons.biom.txt

## Remove non bacterial sequences

mkdir 02_No_Contami

qiime taxa filter-table --i-table 01_No_singletons/asv.table_nosingletons.qza --i-taxonomy ../03_clasif_silva_99/table_casif_99_silva_vsearch.qza --p-exclude Unassigned,Eukaryota,Chloroplast,Mitochondria,Archaea --o-filtered-table 02_No_Contami/asv-table_NoContamination_noSing.qza

qiime tools export --input-path 02_No_Contami/asv-table_NoContamination_noSing.qza --output-path 02_No_Contami/asv-table_NoContamination/

cd 02_No_Contami/asv-table_NoContamination/

biom add-metadata -i feature-table.biom -o asv-table_noCont_noSing.biom --observation-metadata-fp ../../../03_clasif_silva_99/taxonomy_bacteria.tsv --sc-separated taxonomy

biom convert -i asv-table_noCont_noSing.biom -o asv-table_noCont_noSing.tsv --to-tsv --header-key taxonomy --table-type "OTU table"

biom summarize-table -i asv-table_noCont_noSing.biom -o asv-table_noCont_noSing.biom.txt

## Remove low resolution ASVs

cd ../../

mkdir 03_low_res

qiime taxa filter-table --i-table /02_No_Contami/asv-table_NoContamination_noSing.qza --i-taxonomy ../03_clasif_silva_99/table_casif_99_silva_vsearch.qza --p-include p__ --o-filtered-table 03_low_res/asv-table_noCont_noSing_phylo.qza

cd 03_low_res

qiime tools export --input-path asv-table_noCont_noSing_phylo.qza --output-path asv-table_noCont_noSing_phylo/

cd asv-table_noCont_noSing_phylo

biom add-metadata -i feature-table.biom -o asv-table_noCont_noSing_phylo.biom --observation-metadata-fp ../../../03_clasif_silva_99/taxonomy_bacteria.tsv --sc-separated taxonomy

biom convert -i asv-table_noCont_noSing_phylo.biom -o asv-table_noCont_noSing_phylo.tsv --to-tsv --header-key taxonomy --table-type "OTU table"

biom summarize-table -i asv-table_noCont_noSing_phylo.biom -o asv-table_noCont_noSing_phylo.biom.txt

# Obtain final table

qiime tools import --input-path asv-table_noCont_noSing_phylo.biom --type 'FeatureTable[Frequency]' --input-format BIOMV210Format --output-path ../../04_FINAL/asv-table_final.qza

# Rarefaction curves

cd ../../04_FINAL/

qiime feature-table summarize --i-table asv-table_final.qza --o-visualization asv-table_final.qzv --m-sample-metadata-file /path_metadata/metadata.txt

qiime tools view asv-table_final.qzv

qiime diversity alpha-rarefaction --i-table asv-table_final.qza --p-min-depth 24 --p-max-depth 100000 --p-metrics 'observed_features' --o-visualization rarefaction_curves.qzv

conda deactivate

conda deactivate
