# Supplementary material for: Microbiota composition of Culex perexiguus mosquitoes during the West Nile virus outbreak in southern Spain
Source: PLoS One. 2024 Nov 18;19(11):e0314001. doi: 10.1371/journal.pone.0314001 (PMC11573153; doi:10.1371/journal.pone.0314001)
Supplement: S2 File — (DOCX) [file pone.0314001.s006.docx]

####################################################################

##### R SCRIPT FOR THE ANALYSIS OF CULEX PEREXIGUUS MICROBIOTA:#####

##### DIVERSITY AND COMPOSITION #####

####################################################################

# Load packages

library(qiime2R)

library(phyloseq)

library(microViz)

library(ggplot2)

# Load data

physeq<-qza_to_phyloseq(

features="path/features.qza",

taxonomy = "path/taxonomy.qza",

metadata = "path/metadata.txt")

# remove of the feature with ambiguous family annotation

physeq_filt <- subset_taxa(physeq, !is.na(Family) & !Family %in% c("NA", "uncharacterized", "uncultured"))

# Plot microbiome composition by WNV status (Family level)

physeq_filt %>%

tax_names2rank("Family") %>%

comp_barplot(

tax_level = "Family", n_taxa = 10,

bar_width = 0.7, sample_order = rev(c( "D1" , "D2", "D22" ,"D23", "D30" ,"D46" ,"D29" ,"D45", "D47" ,"D48" ,"D49", "D50","D51" ,"D52" ,"D60" ,"D61" ,"D73", "D74","D75" ,"D76", "D78","D79", "D80", "D81",

"D83", "D84" ,"D85" ,"D86" ,"D87" ,"D88", "D89","D90" ,"D91", "D92", "D93" ,"D94",

"D96" ,"D97", "D98", "D99")))

facet_wrap(facets = vars(WNV), scales = "free") +

labs(title = "Relative abundances: Family",

x = "Sample", y = "Relative Abundance") +

coord_flip()

# Analyses at family level

## alpha-diversity

### Richness

rich_family=physeq_filt %>%

ps_calc_richness(rank = "Family", index = "observed", varname = "N_Families")%>%

samdat_tbl()

mean(rich_family$N_Families)

sd(rich_family$N_Families)

### Compare richness between WNV-positive and WNV-negative samples (lm)

physeq_filt %>%

ps_calc_richness(rank = "Family", index = "observed", varname = "N_Families") %>%

samdat_tbl() %>%

lm(formula = N_Families ~ WNV, data = .) %>%

summary()

### Boxplot

(r=RichnessDf %>%

ggplot(aes(y = N_Families, x = WNV, color = WNV)) +

geom_boxplot(alpha = 0.5, width = 0.5, color="black") +

geom_point(position = position_jitter(width = 0.1), alpha = 0.5, size = 2, color="black") +

#scale_color_brewer(palette = "Set1", guide = NULL) +

labs(y = "Observed richness (Family)", x = NULL) +

theme_bw())

### Shannon index

shannon_family=physeq_filt %>%

ps_calc_diversity(rank = "Family", index = "Shannon", varname = "Shannon_Family") %>%

samdat_tbl()

mean(shannon_family$Shannon_Family)

sd(shannon_family$Shannon_Family)

### Compare Shannon index between WNV-positive and WNV-negative samples (lm)

physeq_filt %>%

ps_calc_diversity(rank = "Family", index = "shannon", varname = "Shannon") %>%

samdat_tbl() %>%

lm(formula = Shannon ~ WNV, data = .) %>%

summary()

### Boxplot

(p=ShannonDf %>%

ggplot(aes(y = Shannon, x = WNV, color = WNV)) +

geom_boxplot(alpha = 0.5, width = 0.5, color="black") +

geom_point(position = position_jitter(width = 0.1), alpha = 0.5, size = 2, color="black") +

scale_color_brewer(palette = "Set1", guide = NULL) +

labs(y = "Shannon diversity (Family)", x = NULL) +

theme_bw())

## Beta-diversity

### Principal Coordinates Analysis (PCoA) from jaccard matrix (presence/absence)

(jac_plot= physeq_filt %>%

tax_agg(rank = "Family") %>%

tax_transform("binary") %>%

dist_calc(dist = "jaccard") %>%

ord_calc(method = "PCoA") %>%

ord_plot(alpha = 0.6, size = 2, color = "WNV") +

theme_classic(12) +

coord_fixed(0.7) +

stat_ellipse(aes(color = WNV)) +

scale_color_brewer(palette = "Set1")+

labs(caption = ""))

### PERMANOVA presence/absence 99999 permutations

physeq_filt %>%

tax_agg(rank = "Family") %>%

tax_transform("binary") %>%

dist_calc(dist = "jaccard") %>%

dist_permanova(variables = "WNV", n_perms = 999999, seed = 1234) %>%

perm_get()

### Principal Coordinates Analysis (PCoA) from Bray-curtis matrix (abundances)

(bray_plot= physeq_filt %>%

tax_filter(min_prevalence = 1 / 40, verbose = FALSE) %>%

tax_agg(rank = "Family") %>%

dist_calc(dist = "bray") %>%

ord_calc(method = "PCoA") %>%

ord_plot(alpha = 0.6, size = 2, color = "WNV") +

theme_classic(12) +

coord_fixed(0.7) +

stat_ellipse(aes(color = WNV)) +

scale_color_brewer(palette = "Set1")+

labs(caption = ""))

### PERMANOVA abundances 99999 permutations

physeq_filt %>%

tax_filter(min_prevalence = 1 / 40, verbose = FALSE) %>%

tax_agg(rank = "Family") %>%

dist_calc(dist = "bray") %>%

dist_permanova(variables = "WNV", n_perms = 999999, seed = 123) %>%

perm_get()

## Relative abundances

### Centered Log Ratio transformation

physeq_filt %>%

tax_sort(by = sum, at = "Family", trans = "compositional", tree_warn = FALSE) %>%

tax_agg(rank = "Family") %>%

tax_transform(trans = "clr", zero_replace = "halfmin", chain = TRUE) %>%

comp_heatmap(

samples = 1:40, taxa = 1:15, grid_lwd = 2, name = "CLR",

colors = heat_palette(sym = TRUE),

tax_seriation = "Identity", sample_anno = sampleAnnotation(

WNV = anno_sample_cat("WNV", legend_title = "WNV"))

)

### Obtain taxa relative abundances

py.fam <- tax_glom(physeq_filt, taxrank = "Family")#Agglomerate taxa at family level

py.fam.prop <- transform_sample_counts(py.fam, function(x) x / sum(x) )# Calculate relative abundance

#### EXAMPLE: Family Burkholderiaceae

py.fam.prop.bur <- subset_taxa(py.fam.prop, Family == "Burkholderiaceae")

mean(otu_table(py.fam.prop.bur))

sd(otu_table(py.fam.prop.bur))

### Model all taxon at all levels

shaoModels <- physeq_finalmodel %>%

tax_prepend_ranks() %>%

tax_transform("compositional", rank = "Species", keep_counts = TRUE) %>%

tax_filter(min_prevalence = 0.1, undetected = 0, use_counts = TRUE) %>%

taxatree_models(

type = lm,

trans = "log2", trans_args = list(zero_replace = "halfmin"),

ranks = c("Phylum", "Class", "Order", "Family", "Genus", "Species"),

variables = c("WNV")

)

shaoStats <- taxatree_models2stats(shaoModels)#Stats

shaoStats

shaoStats %>% taxatree_stats_get()

#### p value BH adjustment

shaoStats <- shaoStats %>% taxatree_stats_p_adjust(method = "BH", grouping = "rank")

shaoStats %>% taxatree_stats_get() # Obtain adjusted significance
